# Synchronized extracorporeal shockwave lithotripsy may still affect the heart: a case report of perioperative ST-segment elevation myocardial infarction

**DOI:** 10.3389/fmed.2023.1147725

**Published:** 2023-05-10

**Authors:** Yi Hong Li, Chao Yu Hsu, Chih Tsung Liu, Yi Sheng Lin, Yen Chuan Ou, Min Che Tung

**Affiliations:** ^1^Division of Urology, Department of Surgery, Tungs' Taichung MetroHarbor Hospital, Taichung, Taiwan; ^2^Division of Cardiology, Department of Internal medicine, Tungs' Taichung MetroHarbor Hospital, Taichung, Taiwan

**Keywords:** shockwave lithotripsy, STEMI (myocardial infarction), urolithiasis (urinary stones), complication, case report

## Abstract

Extracorporeal shockwave lithotripsy (ESWL) is widely used as a primary treatment for urolithiasis and is performed as an elective outpatient surgical procedure because of its ease of use. However, patients undergoing this treatment rarely develop cardiac complications. In this article, we present the case of a 45-year-old male patient who presented with ST-elevation myocardial infarction during ESWL. Moreover, atypical symptoms and electrocardiogram patterns were recognized by the nursing staff. Early primary evaluation and intervention resulted in favorable outcomes along with patent coronary artery flow following stent placement for stenosis, and no complications were noted.

## Introduction

Extracorporeal shockwave lithotripsy (ESWL) treats upper urinary tract stones. The European Association of Urology (EAU) guidelines recommend it as a first-line treatment option for proximal/distal ureteral and renal stones of >1 cm and >2 cm, respectively (1). In renal stone management, ESWL demonstrates less tissue damage without any instrument directly entering the renal pelvis; it can be performed as an outpatient surgical procedure unlike retrograde intrarenal surgery and percutaneous nephrolithotomy, which are more invasive. Most patients only need mild sedation and can exhibit symptomatic feedback immediately compared with other operations that require anesthesia (2). However, complications, such as poor stone-free rates, infection, benign arrhythmia, and hematuria, can still occur along with major complications, including perirenal hematoma, bowel perforation, and morbid cardiac events (1). The incidence rate of Clavien III–V complications was 2.5% in a recent meta-analysis of 115 randomized controlled trials (3). In this article, we present the case of a male patient presenting with extended right flank pain during ESWL without symptom relief postoperatively. He had elevated cardiac enzyme levels. His electrocardiogram (ECG) revealed an anterior wall ST-segment elevation myocardial infarction (STEMI). Furthermore, he underwent emergent coronary angiography (CAG), which revealed stenosis of the left anterior descending (LAD) artery, and primary percutaneous coronary intervention (PCI) with stenting for the critical LAD stenosis. He eventually showed improvement without short-term complications.

## Case report

A 45-year-old Asian male patient with renal and ureteral stones underwent bilateral ESWL and flexible laser ureteroscopic lithotripsy for renal stones and bilateral ureteroscopic lithotripsy with LithoClast for ureteral stones several times. He was diagnosed with type 2 diabetes mellitus for >4 years and received saxagliptin plus metformin for blood sugar control. His last HbA1C level was 8.9% within 3 months, and his body mass index was 27.3 kg/m^2^. He had hypertension and chronic gouty arthritis, which were conservatively controlled with medications. His lipid profile revealed that his total cholesterol level (188 mg/dL; reference normal range: 0–200 mg/dL) was within normal limits; however, his low-density lipoprotein (110 mg/dL; normal: 0–100 mg/dL) and triglyceride (239 mg/dL; normal: 0–150 mg/dL) levels were abnormally high, whereas his high-density lipoprotein (27 mg/dL; normal: >40 mg/dL) level was abnormally low. He had no history of cigarette smoking or alcoholism and no family history of coronary artery disease (CAD). He could perform the activities of daily living independently (Canadian Cardiovascular Society angina grade I).

**Figure 1:**
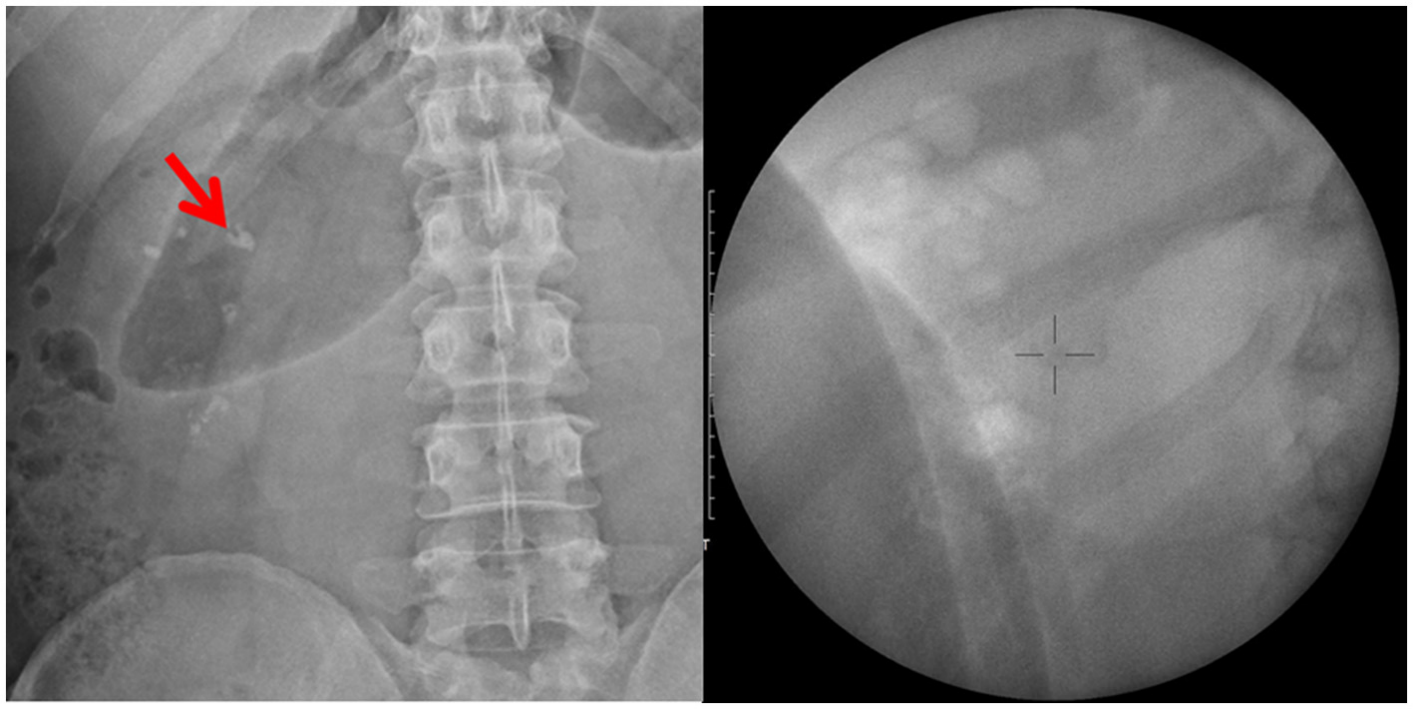
Kidney–ureter–bladder X-ray and fluoroscopy localization before shockwave lithotripsy: right renal stone with a size of 10 × 6 mm.

The patient underwent ESWL again for a right renal stone; however, he developed progressive angina perioperatively. The stone size was approximately 10 × 6 mm on kidney–ureter–bladder X-ray and fluoroscopy scan (Figure 1) at treatment initiation. Bilateral hydronephrosis was ruled out using bedside sonography. Laboratory values were as follows: hemoglobin, 16.6 g/dL; platelet count, 272,000; prothrombin time (PT), 10 s (normal: 8–12 s); activated partial thromboplastin time (aPTT), 26.2 s (normal: 23.9–34.9 s); blood urea nitrogen, 17 mg/dL; creatinine, 1.1 mg/dL; estimated glomerular filtration rate, 76.9 mL/min/1.73 m^2^; hematuria (10–20/HPF); and pyuria (10–20/HPF) in urinalysis. Preoperatively, we prescribed an anesthesia agent with pethidine (25 mg) intravenously. His initial vital signs were as follows: systolic pressure/diastolic pressure, 137/91 mmHg; heart rate, 79 bpm; and SpO_2_, 99%. His vital signs were routinely monitored every 15 min, and the pain was initially tolerated (visual analog scale score of ≤ 2 until 45 min). Subsequently, he complained of extended right flank pain (score = 4). Notably, no desaturation was recorded. The procedure was tentatively paused and started 5 min later. His right flank pain reduced, but he developed chest tightness. Physical examination revealed no flank or abdominal ecchymosis and mild shallow breathing without a paradoxical respiratory pattern. He denied cold sweating or radiating pain to the lower chin or left shoulder. A long lead II ECG test revealed premature ventricular contractions (PVCs) with baseline sinus rhythm. The total number of shocks was 2,940, the total energy was 36.19 J, and the time interval was 75 min.

**Figure 2:**
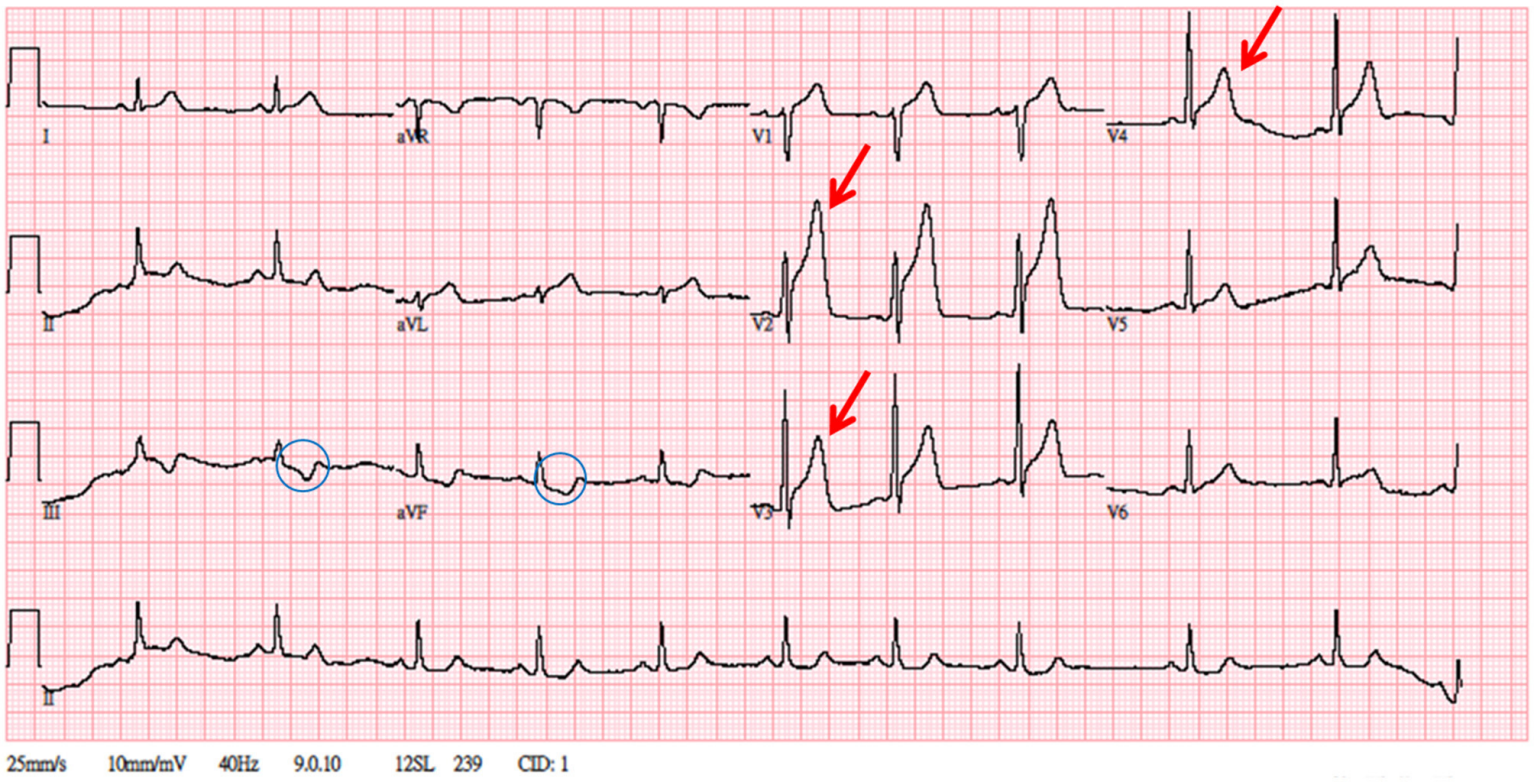
A 12-lead electrocardiogram. Significant ST-segment elevation over V1–V4, hyperacute T over V2–V3, and reciprocal change over leads III and aVF. The patient's troponin level was elevated to 0.239 ng/mL.

**Figure 3:**
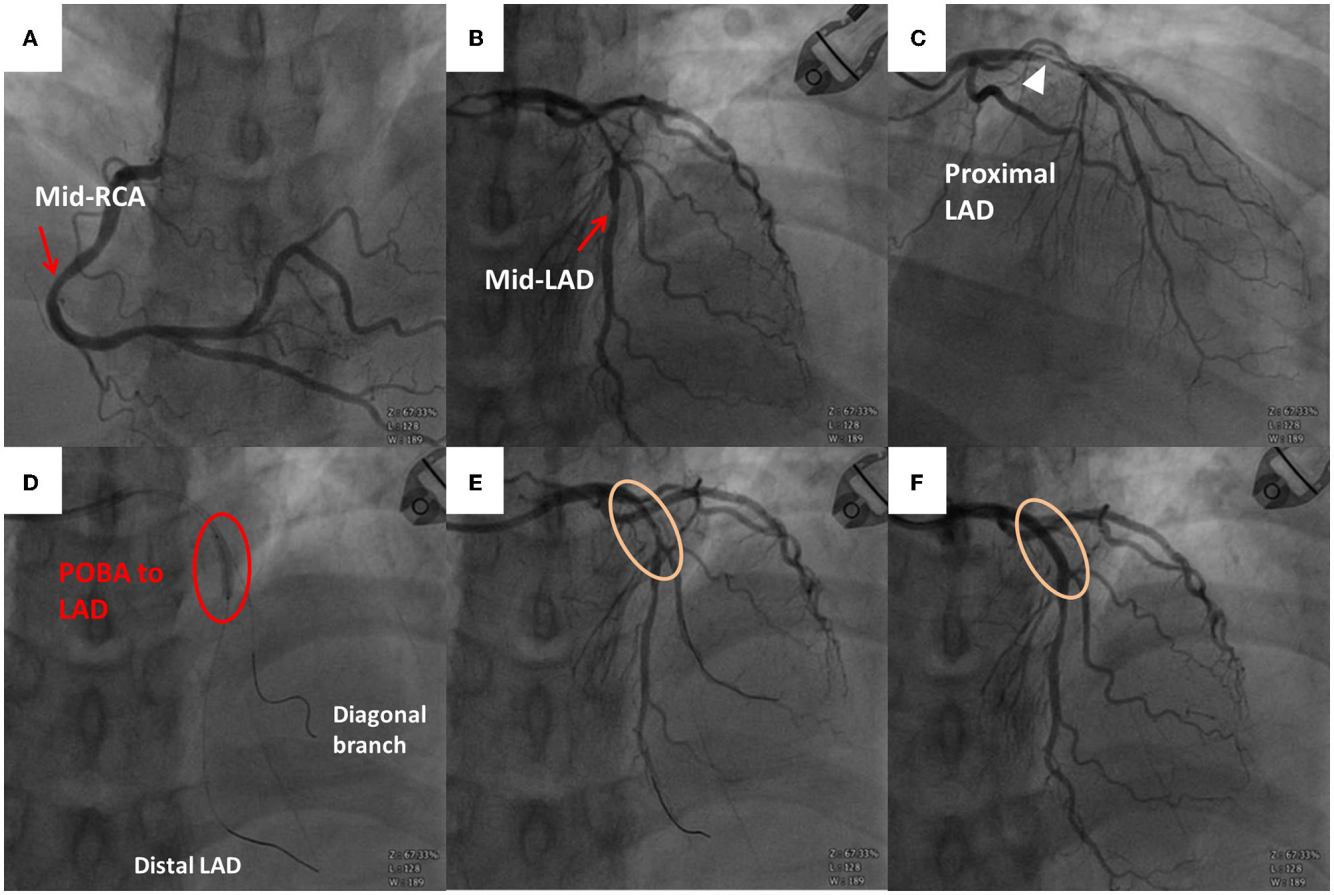
Emergent coronary angiography (CAG) scan. **(A)** Right coronary artery with stenosis over the middle segment (red arrow). **(B)** Left anterior descending (LAD) artery with stenosis over the middle segment (red arrow). **(C)** Severe stenosis over the proximal LAD artery (white triangle). **(D)** Plain old balloon angioplasty (POBA) to the proximal LAD artery. Guidewire within the distal LAD artery and diagonal branch. **(E)** Follow-up angiography after the first POBA with residual stenosis. **(F)** Follow-up angiography after the second POBA and stent implantation (Orsiro 3.0 × 30 mm) without residual stenosis over the proximal LAD artery.

The patient was referred to the emergency department for further examinations. Point-of-care ultrasound was negative for bilateral perirenal hematoma or fluid collection in the Morison pouch or cul-de-sac. The diameter of the abdominal aorta was within the normal range. A 12-lead ECG revealed a hyperacute T wave with ST-segment elevations in leads V1–V4 and reciprocal ST-segment depressions in leads III and aVF (Figure 2). Postoperative laboratory results were as follows: hemoglobin, 16.2 g/dL; platelet, 237,000; PT, 10.3 s; and aPTT, 23.1 s. Regarding cardiac enzymes, troponin-I, creatine kinase (CK), and CK-myoglobin binding (CK-MB) levels were <0.02 ng/mL (normal: <0.02 ng/ml), 169 U/L (normal: 45–163 U/L), and 26.4U/L (normal: <24 U/L), respectively; however, subsequently, troponin-I and CK levels increased to 0.239 ng/mL and 204 U/L, respectively. Emergent CAG and primary PCI were scheduled for anterior wall STEMI. As pre-PCI medications, aspirin (300 mg), ticagrelor (180 mg), and unfractionated heparin (loading dose, 4,000 IU) were administered. The patient had 50% mid-right coronary artery stenosis, 50% mid-LAD stenosis, and 90% proximal LAD stenosis (Figures 3A–C). A grade 2 distal LAD TIMI flow was observed (contrast perfusion of the entire artery but delayed flow). The left circumflex artery was patent. A type B complexity lesion was interpreted based on the American College of Cardiology/American Heart Association classification. Plain old balloon angioplasty was performed, and one drug-eluting stent (Orsiro 3.0 × 30 mm) was implanted at the proximal LAD to restore TIMI 3 flow (full perfusion and normal flow) (Figures 3D–F). The procedure lasted for 62 min, and he was transferred to the intensive care unit. His vital signs remained stable without any associated signs and symptoms. A decrease was observed in troponin-I (0.038 ng/mL), CK (181 U/L), and CK-MB (19.2 U/L) levels. Follow-up ECG revealed the following: ST elevation reduced to an isoelectric line; no T wave inversion; and only a Q wave in lead II. Furthermore, an echocardiogram revealed concentric left ventricle hypertrophy; however, relative hypokinesia at the anterior wall of the left ventricle was also observed. Additionally, the left ventricle contractility was fair with preserved left ventricular systolic function (ejection fraction: 60%). The patient was transferred to the general ward on day 3 and discharged on day 4. Aspirin and ticagrelor (antiplatelet agents), bisoprolol (a beta-blocker), aliskiren (a renin inhibitor), atorvastatin (a statin), and sodium–glucose cotransporter 2 inhibitor plus dipeptidyl peptidase 4 inhibitor (oral hypoglycemic agents) were prescribed after the procedure and continued even after his discharge. The patient denied any associated signs and symptoms in a series of outpatient follow-ups.

## Discussion

ESWL is a treatment option for urolithiasis. Candidate eligibility for this procedure depends on the stone burden, location, and composition. The EAU guidelines suggest ESWL for renal stones of <20 mm, except for the lower pole stones owing to their poor stone-free rates (1). Moreover, ESWL is contraindicated in pregnancy, bleeding disorders, uncontrolled urinary tract infection, severe skeletal deformities, arterial aneurysms, and anatomical obstruction distal to the stone (1).

ESWL complications can be classified into those related to stone fragments, infections, and tissue effects. Regarding tissue effects, ESWL is most commonly related to cardiovascular effects, followed by renal and gastrointestinal effects. After lithotripsy with synchronous mode was introduced, this lithotripsy became the standard modality for decreasing the incidence of arrhythmias (4). The ECG lead is patched; synchronization is started when the cardiac cycle is recognized; and then, shockwave release is avoided during the relative refractory period. The incidence rate of arrhythmias under synchronized ESWL is 11%−59%, whereas, under unsynchronized ESWL, it is 60%−80%; however, arrhythmias during synchronized ESWL are mostly benign and do not require intervention (5). Unifocal PVCs were mostly recorded (4, 6); multiple PVCs and supraventricular tachycardia have also been reported. Greenstein et al. analyzed the risk of developing arrhythmias in 125 patients (6). Arrhythmias were commonly related to the right-sided procedures (*p* < 0.05) but were not related to age, sex, pre-existing heart disease, stone size, location, ureteral stent presence, anesthesia mode, and total shock number. Thomas et al. reported similar outcomes in 342 patients and found that arrhythmias were common in younger patients and right-sided procedures (5).

It is believed that there is a proportional injury to the surrounding organs because shockwaves cannot be solely targeted to the kidney or ureter. Myocardial injury during ESWL was evaluated. Different biomarkers, including cardiac enzymes, were mostly used during or after the procedure, and CK was first used. Kirk et al. found significantly elevated CK levels after ESWL, with pre-ESWL CK as the control (*p* < 0.001) (7). However, there was no correlation among symptoms, ECG changes, and CK levels. They suggested that the CK levels were elevated due to skeletal muscle injury as no elevation in CK-MB levels was observed in all the patients. Regarding troponin levels, no elevation was reported after blood sample collections within 5 min (8) or after 24 h (9).

Preprocedure analgesics may mask myocardial infarction (MI) symptoms. Morphine is the only recommended analgesic and can be used in some patients with chest pain, dyspnea, and anxiety as per the European Society of Cardiology guidelines for STEMI management (10). The efficacy of pethidine was compared with that of morphine. Some studies have demonstrated similar pain relief efficacy in patients with MI between the pethidine and morphine groups. However, Solhi et al. reported poor pain control in the pethidine group (11). Even though pethidine is not a recommended MI drug, it may help diagnose difficulties due to analgesia.

We understand that our patient had a cardiac event without pre-existing CAD. However, the potential mechanism of this phenomenon remains unclear. MI can be divided into five classes. Type I is the most common with vulnerable plaque rupture or endothelial ulceration, and type II is characterized by supply–demand imbalance based on the presence of stable plaques. Perioperative MI (PMI) was rarely accessed before the 21st century (12) but became widely known after the introduction of troponin. Troponin (cardiac enzyme) has a higher sensitivity for predicting myocardial injury than CK and CK-MB. Moreover, it is not affected by skeletal muscle injury but is sometimes elevated in sepsis, renal insufficiency, and pulmonary embolism. Type 2 MI (T2MI) has features similar to type 1 MI; however, most individuals with T2MI present with non-ST-segment elevation, whereas, only 3%−24% have STEMI on ECG (13). PMI is usually silent, and the ECG changes are often transient or have insignificant ST–T changes, which are the leading causes of postoperative mortality (14). The pathophysiology of PMI is incompletely understood. T2MI may be predominantly assessed in the non-cardiac surgery MI review (15).

ESWL is a seldom discussed low-risk surgery because the aforementioned studies only focused on non-cardiac major surgeries. The procedure is usually performed while the patient is awake, allowing the patient to provide immediate feedback in case of discomfort. Moreover, precipitating factors can be eliminated or reduced tentatively by technicians who perform the procedure. The procedure may also decrease the supply–demand imbalance possibilities.

We also hypothesize that there may be some time dependence between CAD and stone cases, which may explain why our patient did not develop cardiac complications previously. In our case, type 2 DM was a risk factor for CAD, but further evidence indicated urolithiasis. Cheungpasitporn et al. reported that patients with renal stones had a relative risk (RR) of 1.24 for developing CAD; however, it was significant in female patients (RR, 1.43; 95% confidence interval [CI], 1.12–1.82) but not in male patients (RR, 1.14; 95% CI, 0.94–1.38) (16). Conversely, Peng et al. reported an RR of 1.23 between renal stones and CAD, which was significant in male patients (RR, 1.23; 95% CI, 1.02–1.49) but not in female patients (RR, 1.01; 95% CI, 0.92–1.11) (17). Both results showed that renal stones were proportional to CAD, although there were some conflicts between the two meta-analyses.

## Conclusion

This case represents a morbid cardiac complication in a low-risk ESWL urological surgery. The patient could exhibit symptomatic feedback owing to the light sedation, unlike those undergoing other procedures that require anesthesia. However, the patient's outcomes were still dependent on the urologists' and team members' alertness. Despite the rarity of the condition, urologists should pay more attention to atypical symptoms or discomfort that are generally not experienced by individuals with underlying type 2 DM or stone.

## Data availability statement

The raw data supporting the conclusions of this article will be made available by the authors, without undue reservation.

## Ethics statement

Ethical review and approval was not required for the study on human participants in accordance with the local legislation and institutional requirements. Written informed consent from the patients/participants or the patients'/participants' legal guardian/next of kin was not required to participate in this study in accordance with the national legislation and the institutional requirements. Written informed consent was obtained from the patient for publication of this case report and any accompanying images.

## Author contributions

YLi was responsible for the conception and design of the study, the analysis and interpretation of the collected data, and drafted the article. CH and CL participated in the care of the patient. All authors read and approved the final version of the manuscript to be submitted.
